# Comment on: Target-based discovery of a broad-spectrum flukicide

**DOI:** 10.1371/journal.pntd.0012656

**Published:** 2024-11-27

**Authors:** Timir Tripathi

**Affiliations:** Molecular and Structural Biophysics Laboratory, Department of Zoology, North-Eastern Hill University, Shillong, India; Research Center for Hydatid Disease in Iran Kerman University of Medical Sciences, ISLAMIC REPUBLIC OF IRAN

Triclabendazole (TCBZ) is used as a highly potent drug against the liver flukes of the genus *Fasciola*. It acts by disrupting microtubule formation and interfering with energy metabolism. Its targeted and specific action against these parasites makes it an excellent candidate for treating fascioliasis. However, emerging reports indicate a growing resistance to TCBZ among *Fasciola* populations. In contrast, praziquantel (PZQ) is ineffective against *Fasciola* due to a single amino acid alteration in the target protein, a transient receptor potential ion channel of the melastatin family (TRPM_PZQ_) within these species [1,2]. A recent study by Marchant and colleagues [3] has identified a new broad-spectrum drug, a benzamidoquinazolinone analog (BZQ), which successfully activates TRPM_PZQ_ ion channels across various parasitic flukes, including *F*. *hepatica*, a species resistant to PZQ. The discovery of BZQ represents a significant breakthrough as it serves as a potent, broad-spectrum activator of TRPM_PZQ_ ion channels in parasitic flukes. Notably, BZQ was shown to be effective against both *S*. *mansoni* and *F*. *hepatica* in *ex vivo* studies, inducing rapid paralysis and causing tegumental damage similar to the effects of PZQ on schistosomes. Additionally, BZQ demonstrated efficacy comparable to PZQ in reducing worm burden in a mouse model of schistosomiasis. The development of BZQ could pave the way for a new, effective treatment for trematode infections that have been resistant to current therapies, including TCBZ.

## Target-based discovery of a broad-spectrum flukicide

Quinazolinones are known for their diverse biological activities, including antibacterial, antifungal, and anticancer properties. The authors identified that one of their 22 compounds (an *N*-benzamidoquinazolinone) is a potent agonist for SmTRPM_PZQ_ (EC_50_ = 1.15 μM) and FhTRPM_PZQ_ (EC_50_ = 3.0 μM), highlighting its effectiveness against both these targets. To further optimize the activity of the *N*-benzamidoquinazolinone scaffold, a structure–activity relationship (SAR) analysis was conducted. A series of PZQ analogs were synthesized to explore SAR, revealing stringent requirements for efficacy at TRPM_PZQ_. Among these, compound 20 emerged as the most potent agonist, with an EC_50_ of 0.09 μM for SmTRPM_PZQ_. Electrophysiological recordings confirmed that BZQ is a more potent activator of both SmTRPM_PZQ_ and FhTRPM_PZQ_ compared to compound 1, with EC_50_ values of 0.11 μM and 0.27 μM, respectively. BZQ induced single-channel activity in both TRPM_PZQ_ variants, demonstrating its ability to activate the channels effectively. Notably, BZQ caused rapid contraction and paralysis in *S*. *mansoni*. At the same time, it effectively induced muscle contraction and surface damage in *F*. *hepatica*, unlike PZQ, which showed no effect on the liver fluke. BZQ exhibited high potency against various *Fasciola* species, with IC_50_ values of 1.12 μM for immature and 2.72 μM for adult *F*. *hepatica* in motility assays, indicating its potential as a more effective treatment option. Computational modelling studies revealed that BZQ engages the TRPM_PZQ_ binding pocket similarly to PZQ [4–6] but exploits a conserved threonine residue, allowing it to activate both SmTRPM_PZQ_ and FhTRPM_PZQ_ effectively. Experimental validation through point mutations confirmed the predicted interactions, underscoring the broad-spectrum activity of BZQ across fluke TRPM_PZQ_ orthologs.

## Importance of the work for treating fluke infections

The work is particularly important as it targets trematode infections, specifically flukes, which cause significant diseases in humans, such as schistosomiasis and fasciolosis. Schistosomiasis affects over 250 million people globally [7], while fasciolosis impacts an estimated 2.6–17 million people worldwide, primarily in rural areas. In addition to being a major concern for human health, fasciolosis poses a significant threat to livestock, resulting in substantial economic losses in agriculture [8]. Current treatment options like PZQ and TCBZ have limitations, including ineffectiveness against certain fluke species and the emergence of drug resistance [9,10]. For instance, PZQ is ineffective against liver flukes from the genus *Fasciola* [11,12]. The design of new inhibitors targeting the catalytic mechanism of key proteins in these parasites is a complex process [13]. The development of new drugs like BZQ addresses this gap, as it shows promise against multiple fluke species, including those resistant to existing treatments. This broad-spectrum efficacy is essential for improving treatment outcomes and controlling fluke-based diseases in diverse populations. Given that many trematode infections affect both humans and livestock, the development of BZQ also has veterinary applications. This dual utility can help control infections in animal populations, which in turn can reduce transmission to humans and improve overall public health.

However, the work also has certain limitations. The authors did not perform a selectivity test to evaluate whether BZQ activates human TRPM8 or other human TRPM channels in the HEK293 cell line despite the presence of conserved key residues for compound interaction among parasitic and human channels. This could have provided insights into the potential off-target effects of BZQ in humans. The *in vivo* testing of BZQ and PZQ in a mouse model of schistosomiasis was conducted via intraperitoneal injection. This method of administration does not align with the current treatment protocol for PZQ and is not suitable for such a drug. Testing the effectiveness of BZQ through oral administration would have been more relevant for its potential future applications.

## Application of current research in treating trematode infections in humans

The identification and optimization of BZQ, which shows promising efficacy against *F*. *hepatica*, is particularly significant as PZQ, the standard treatment for many trematode infections, is ineffective against these parasites. The broad-spectrum activity of BZQ suggests that it could potentially treat multiple trematode infections in humans, addressing a critical gap in current treatment options. The emergence of drug resistance, particularly to TCBZ in liver flukes, highlights the urgent need for new therapeutic options. BZQ offers a viable alternative that may be effective against resistant strains, thereby improving treatment outcomes for patients suffering from trematode infections. Furthermore, BZQ has demonstrated significant efficacy not only *in vitro* but also *in vivo*, as evidenced by its performance in murine models. This suggests that BZQ could be a potential therapeutic agent against trematode infections in humans, particularly in regions where these infections are endemic. The ability of BZQ to universally activate TRPM_PZQ_ across various fluke species positions it as a promising first-in-class flukicide. This broad-spectrum activity is particularly valuable in regions with multiple prevalent fluke infections, as it enables a single therapeutic agent to simultaneously target more than one fluke-based disease. Such an approach could simplify treatment regimens and enhance patient compliance. These findings align with global health initiatives focused on controlling and eliminating neglected tropical diseases (NTDs), including the WHO’s goal of eliminating schistosomiasis as a public health problem by 2030. This work supports the broader objective of reducing the incidence and impact of NTDs by introducing a new treatment option for fluke infections.

## Future research directions

Key questions for research investigations that need to be addressed include:

- How effective is BZQ against other fluke species or different life cycle stages, and does its efficacy extend beyond *F*. *hepatica* and *S*. *mansoni* for broader-spectrum treatment?
- What is the detailed three-dimensional structure of the BZQ-TRPM_PZQ_ complex, and what are the exact molecular mechanisms of their interaction, as well as the underlying basis of its therapeutic action?
- Given that BZQ is a new compound, what thorough assessments are required to evaluate potential off-target effects and toxicological concerns to ensure its safety for widespread use in both human and veterinary medicine?
- While BZQ shows promising *in vivo* antischistosomal activity comparable to PZQ, comprehensive animal studies are needed to fully understand its pharmacokinetics, pharmacodynamics, and safety profile before it can be considered safe for clinical use.
- Additionally, considering that some studies have reported the toxicity of certain quinazolinone derivatives to mammalian cells if required [14,15], what specific structural modifications can be made to these derivatives to minimize their cytotoxic effects while at the same time preserving their therapeutic efficacy?
- What are the risks and potential for resistance development in target parasites when treated with BZQ, and how can resistance patterns be effectively monitored to ensure the long-term sustainability of its effectiveness?
- How can a deeper understanding of these mechanisms facilitate the development of more effective and safer derivatives of BZQ?

Ultimately, it is up to the scientific community to critically assess whether there is a genuine need for the development of additional chemicals to combat animal parasites in the long term. Additionally, it is essential to consider whether the use of the same compounds in both animals and humans could contribute to an increased risk of drug resistance. Addressing these questions will be essential for advancing the development of BZQ as a reliable broad-spectrum anti-trematode treatment and ensuring its safe, effective, and sustainable use in the future.
